# Aortic Valve and Left Ventricular Outflow Tract Calcification as Predictors of Early Complications After Transcatheter Aortic Valve Implantation: A Prospective Single-Center Study

**DOI:** 10.14740/cr2247

**Published:** 2026-07-17

**Authors:** Abubakr Muhamed Bakr, Mansour Mohamed Mostafa, Mohamed Adel Attia, Ahmad E. Mostafa, Diaa Kamal, Ayman Ahmed Gad, Waseem Omar Ahmed, Sariya Khan, Muhammad Saad Reihan

**Affiliations:** a Cardiology Department, Faculty of Medicine, Al-Azhar University, New Damietta, Egypt; b Cardiology Department, Faculty of Medicine, Al-Azhar University, Cairo, Egypt; c Cardiology Department, Faculty of Medicine, Ain Shams University, Cairo, Egypt; d General Medicine Practice Program, Batterjee Medical College, Jeddah 21442, Saudi Arabia; e Department of Internal Medicine, General Medicine Practice Program, Batterjee Medical College, Jeddah 21442, Saudi Arabia

**Keywords:** Calcification, Left ventricular outflow tract calcification, Transcatheter heart valve, Transcatheter aortic valve implantation

## Abstract

**Background:**

The prognostic implications of aortic valve and left ventricular outflow tract (LVOT) calcification following transcatheter aortic valve implantation (TAVI) are uncertain. The aim of the study was to assess the influence of aortic valve and LVOT calcification on early TAVI outcomes.

**Methods:**

From October 2021 to May 2024, in this single-center prospective cohort study, preoperative aortic valve calcium score via the Agatston method was determined by multidetector computed tomography (MDCT), and LVOT calcification was semi-quantitatively assessed in 95 consecutive symptomatic severe aortic stenosis patients who underwent TAVI. Primary endpoints assessed within 30 days after TAVI were paravalvular leak (PVL > grade 2), permanent pacemaker implantation (PPM) for high-grade atrioventricular (AV) block, and requirement for postimplantation balloon dilatation.

**Results:**

The mean calcium score was 2,658 ± 1,799. Five patients (5.2%) required PPM, and 14 (14.7%) developed transient PVL > grade 2, all of which improved after dilatation. The aortic valve calcium score was associated with PVL (area under the curve (AUC) = 0.70; P < 0.01), high-grade AV block (AUC = 0.66; P = 0.02), and postimplantation dilatation (AUC = 0.64; P = 0.03). LVOT calcification predicted high-grade AV block (P = 0.027).

**Conclusions:**

Quantitative LVOT and aortic valve calcification grading is a reliable predictor of early conduction and valvular dysfunction complications after TAVI and demonstrates the value of comprehensive preprocedural computed tomography (CT) imaging for procedural planning and risk stratification.

## Introduction

Severe aortic stenosis (AS) is a condition of high mortality and morbidity unless it is repaired. Surgical aortic valve replacement (SAVR) has been the favored treatment for severe symptomatic AS regardless of etiology of the stenosis [1, 2]. However, highly-risk surgical patients may be denied their curative treatment because of high postoperative complications and mortality. Therefore, a new, less invasive and safer aortic valve replacement technique is in dire need [3]. In 2002, Cribier et al implanted the first transcatheter heart valve (THV) with antegrade technique via femoral venous circulation as a point of access. Subsequently, through transseptal methods, they successfully implanted their valve in the position of aorta [4, 5].

Following the primary procedure, more than 1.5 million transcatheter aortic valve implantation (TAVI) procedures have been performed with different techniques and different platforms (THV). Subsequent TAVI trials have also explored its use in lower-risk patients and young patients to extend the benefits of this less invasive and safer procedure to broader patient populations [6]. In spite of increased TAVI acceptance, some procedural success and complications are still affected by specific anatomical features.

Aortic valve and left ventricular outflow tract (LVOT) calcification are collectively referred to as the landing zone and can negatively influence device expansion, sealing, and conduction channels. Severe calcification has also been linked to paravalvular leak (PVL), conduction defects, and balloon dilatation or malpositioning risk of the device [7]. However, most of the previous research have noted total aortic valve calcium score, with less attention on LVOT calcification and its direct contribution to early outcomes. Calcification burden and pattern in LVOT and aortic valve can individually and synergistically contribute to the formation of procedural complications, but evidence evaluating their combined impact remains limited, particularly from more recent valve platforms such as the Evolut R and Sapien 3 platforms [8]. Furthermore, standardized cutoffs according to quantitatively derived criteria for individual adverse outcomes, such as high-grade atrioventricular (AV) block or gross PVL, are not well defined.

Therefore, this study aimed to assess the predictive role of aortic valve and LVOT calcification by Agatston method and visual grade on multidetector computed tomography (MDCT) for early TAVI complications, including conduction disturbances, paravalvular leakage, and postimplantation balloon dilatation.

Although there are enough data which indicate that aortic valve and LVOT calcification are factors that can predict adverse outcomes after TAVI, it is also clear that there is not enough information available on the issue yet. Previous studies have focused on the examination of either total calcium burden of the valve or distribution of leaflet calcification, and the sample of valves in question was heterogeneous. In our research, we explored both quantitative calcium burden of the aortic valve and LVOT calcification in a relatively homogeneous cohort treated predominantly with Evolut R valves and examined their connection to a few clinical complications.

## Materials and Methods

### Study design and setting

This prospective, single-arm, observational trial was conducted at the Nasr City Health Insurance Hospital, Egypt, between October 2021 and May 2024. The protocol for this study conformed to the Declaration of Helsinki (2013 revision) and was approved by the Institutional Ethics Committee (IEC) in Al-Azhar University, Cairo, Egypt (IEC Approval No.: 0000150).

### Study population

This single-arm observational study enrolled all symptomatic severe AS patients who were presented for TAVI evaluation from October 2021 to May 2024. Consecutive sampling was utilized to circumvent selection bias where all such patients who happened to come under the eligibility criteria during these dates were enlisted. Inclusion criteria were echocardiographically characterized severe symptomatic AS defined by an aortic valve area (AVA) of ≤ 1.0 cm^2^, mean transvalvular gradient ≥ 40 mm Hg, or peak velocity ≥ 4.0 m/s, and patients were considered to be appropriate candidates for TAVI by the institutional heart team. Suitability by MDCT and clinical appropriateness were evaluated. Only intermediate-risk or high-risk surgery patients, as categorized by the European System for Cardiac Operative Risk Evaluation II (EuroSCORE II) or Society of Thoracic Surgeons (STS) score, were included.

Exclusion criteria comprised of persons younger than 65 years old and SAVR patients who declined written informed consent or had contraindications to contrast-enhanced computed tomography (CT) scan, such as severe renal impairment or extensive arrhythmia leading to suboptimal image quality. Moreover, patients with contraindication to anatomical TAVI, a life expectancy less than 1 year, or not indicated according to the heart team based on futility were excluded. Patients who died during hospitalization or were lost to follow-up were excluded from the final per-protocol analysis. Thirty patients were excluded for these reasons among the 127 patients screened, and 97 received TAVI. Two of them died during the index hospitalization, and 95 were included in the final study analysis.

### Preprocedural evaluation

Preprocedure workup of all the patients listed was performed in the form of detailed clinical history (past history of cardiac surgery, comorbid illness, age, and gender), adequate physical examination, and baseline investigation. Baseline 12-lead electrocardiogram was done for the assessment of PR interval, QRS duration, and conduction abnormality assessment at baseline. Transthoracic echocardiogram (TTE) was used to assess AVA, mean and peak pressure gradient, ejection fraction (EF), size of the left ventricle, and severity of aortic and mitral regurgitation. MDCT was also used in all patients to assess the anatomy of aortic root, size of the annulus, location of coronary ostia, form of the ascending aorta, iliofemoral access pathway, and to assess the density of calcification of the aortic valve leaflets and LVOT.

### Assessment of aortic valve and LVOT calcification

Quantitative as well as qualitative assessment were used in calcification analysis. Calcium load of the aortic valve was quantitated on non-contrast CT imaging with the Agatston score, and morphologic grading was visually estimated and graded across four categories according to degree of calcific involvement: grade 1 (tip calcification, mild), grade 2 (leaflet body involvement, moderate), grade 3 (dense commissural calcification and fusion), and grade 4 (dense calcification beyond annulus). Calcification of the LVOT was visually scored on CT as present or absent, and when present, its location (anterior, posterior, septal) was recorded. The thickness of calcification was measured by two experienced observers in blind reporting to limit variation in subjectivity and, where there was disagreement, consensus was reached.

### Procedural characteristics

All interventions were performed by a senior multidisciplinary TAVI team under conscious sedation or general anesthesia in keeping with standard institutional practice. Two THV devices were used: self-expandable Evolut R (Medtronic, USA) in 93 patients (97.9%) and balloon-expandable Sapien XT/Sapien 3 (Edwards Lifesciences, USA) in two patients (2.1%). The access route (surgical or percutaneous, femoral or subclavian approach), pre- or post-dilatation balloon dilatation, and valve type were decided based on preprocedure imaging and operator choice. Implant depth and other procedural details were recorded in all the patients. The main endpoints of concern were early complications after TAVI during the 30-day interval after the procedure. Membranous septum length was measured on preprocedural CT according to standard methodology.

### Outcome measures

These were AV block of high-grade requiring permanent pacemaker implantation (PPM), moderate-to-severe PVL (> grade 2), and requirement for post-implant balloon dilatation. Secondary events comprised new conduction abnormalities (left bundle branch block (LBBB), QRS widening, PR prolongation), cardiovascular events (myocardial infarction and cerebrovascular accidents), and transvalvular residual pressure gradients at device implant. All the events were prospectively defined and classified according to Valve Academic Research Consortium-3 (VARC-3) to enable direct correlation and comparison with TAVI published data.

### Ethical considerations

All participants were informed, and informed consent was obtained. Abiding by all ethical principles, the information collected was strictly kept confidential by employing secure data management protocols. The approval of the study was acquired by IEC, to make sure that the study is compliant with guidelines relevant to the ethical standards. The entirety of the data collected was kept confidential by using encryption methods, allowing access only to the investigators.

### Statistical analysis

Continuous variables were presented as means with standard deviations (SD). Categorical variables were expressed as frequencies and percentages. To compare differences between groups, we used Student’s *t*-test for continuous variables and the Chi-square test for categorical variables. Diagnostic analysis included the calculation of the area under the receiver operating characteristic (ROC) curve (AUC) to evaluate the predictive accuracy of calcium score in post-TAVI complications or the need for pre- or post-dilatation. Correlation analysis was conducted using Pearson or Spearman correlation coefficients to assess the relationships between different variables and adverse outcomes in all patients. Statistical significance was defined as a P value less than 0.05. All statistical analyses were performed using SPSS and R programming with RStudio (Build 402).

## Results

In our study, 127 patients with symptomatic severe AS were referred to our centers for eligibility assessment for TAVI. Thirty patients were excluded according to our exclusion criteria; 97 patients underwent TAVI. Two patients died during hospital stay, and the remaining 95 patients were available for 1-month follow-up and included in the final analysis.

### Baseline and pre-procedural characteristics

The mean age of the population was 73.1 ± 6.0 years, and the majority of patients were men (61.1%). The mean body mass index (BMI) was 30.9 ± 6.1 kg/m^2^, and the mean EuroSCORE II was 0.95±1.82%, which showed that most of the patients belonged to the intermediate surgical-risk category. The most common cause of tricuspid AS was degenerative in the majority of the patients, with 16 (16.8%) and 10 (10.5%) patients having bicuspid and rheumatic disease, respectively. Baseline comorbid conditions included hypertension, diabetes mellitus, ischemic heart disease, and history of coronary artery bypass grafting (CABG). Baseline electrocardiogram and echocardiogram results are shown in Tables 1 and 2. Left ventricular EF was 60.8±12.1%, and transvalvular pressure gradient was 53.4 ± 15.3 mm Hg on average. Median membranous septum length was 9 mm (range 4–14 mm).

**Table 1:** Baseline Data of Studied Patients (N = 95)

Variables	Results
Age (years)	73.144 ± 5.998
Sex	
Male	58 (61.05%)
Female	37 (38.9%)
Weight (kg)	82.979 ± 14.951
Height (cm)	163.854 ± 8.513
BMI (kg/m^2^)	30.930 ± 6.080
BSA (m^2^)	1.895 ± 0.181
EuroScore II, %	0.951 ± 1.823
CrCl (mL/min)	87.016 ± 29.158

Data expressed as mean (SD), frequency (percentage). BMI: body mass index; BSA: body surface area; CrCl: creatinine clearance; EuroSCORE II: European System for Cardiac Operative Risk Evaluation II; SD: standard deviation.

**Table 2:** Preoperative ECG, Echocardiographic Parameters Among the Studied Patients (N = 95)

Variables	Results
AF	
No	84 (88.4%)
Permanent	5 (5.2%)
Paroxysmal	6 (6.3%)
BBB	
No	82 (86.3%)
LBBB	6 (6.3%)
RBBB	7 (7.3%)
PR interval duration (ms)	154.6 ± 55.4
QRS duration (ms)	89.8 ± 17.7
EF (%)	60.813 ± 12.151
SWT (mm)	13.542 ± 1.615
PWT (mm)	13.031 ± 1.209
LVEDD (mm)	50.802 ± 7.969
LVESD (mm)	33.349 ± 7.886
PPG (mm Hg)	85.900 ± 23.057
MPG (mm Hg)	53.444 ± 15.334
AVA (cm)	0.750 ± 0.157
RVSP (mm Hg)	40.896 ± 12.479
AR	
0	0 (0%)
1	71 (74.7%)
2	18 (18.9%)
3	5 (5.2%)
4	1 (1%)

AF: atrial fibrillation; BBB: bundle branch block; LBBB: left bundle branch block; RBBB: right bundle branch block; EF: ejection fraction; SWT: septal wall thickness; PWT: posterior wall thickness; LVEDD: left ventricular end diastolic diameter; LVESD: left ventricular end systolic diameter; PPG: photoplethysmography; MPG: mean pressure gradient; AVA: aortic valve area; RVSP: right ventricular systolic pressure; AR: aortic regurgitation.

### CT findings

The mean annular diameter was 23.8 ± 2.6 mm, and the mean annular perimeter was 77.3 ± 7.8 mm. The mean aortic valve calcium score, according to the Agatston method, was 2,658 ± 1,799. Valve calcification was visually graded grade 1 in 13.6% of patients, grade 2 in 20.8%, grade 3 in 34.4%, and grade 4 in 30.2%. Calcification of LVOT was identified in 17 (17.9%) patients, most commonly at septal margin (n = 10, 58.8%), followed by posterior (n = 5, 29.4%) and the diffuse type (n = 2, 11.8%) as shown in Table 3. The agreement between two independent observers for valve calcification grading and LVOT was optimum (κ = 0.87).

**Table 3:** Preoperative CT Parameters Among the Studied Patients (N = 95)

Variables	Results
Annulus diameter (mm)	23.81 ± 2.55
Annulus perimeter (mm)	77.35 ± 7.75
LMCA (mm)	12.98 ± 2.33
RCA (mm)	16.02 ± 2.87
MS (mm)	9 (4–14)
Grade of aortic valve Calcification	
1	13 (13.6%)
2	20 (20.8%)
3	33 (34.4%)
4	29 (30.2%)
LVOT calcification	17 (17.7%)
Septal bulge	22 (23.15%)
RHD	10 (10.5%)
BAV	16 (16.8%)
Overall horizontal aorta	20 (20%)
Horizontal aorta in bicuspid aortic valve	3 (15%)

LMCA: left main coronary artery; RCA: right coronary artery; MS: mitral stenosis; LVOT: left ventricular outflow tract; RHD: rheumatic heart disease; BAV: bicuspid aortic valve

### Procedural features

All the patients were successfully implanted with TAVI. Self-expanding Evolut R valve (Medtronic, USA) was implanted in 93 patients (97.9%), and two patients (2.1%) received the balloon-expandable Sapien XT/Sapien 3 (Edwards Lifesciences, USA) device. Valve sizes of 29, 34, 26, and 23 mm were implanted in 45 (46.9%), 24 (25.2%), 24 (25.0%), and two (2.1%) patients, respectively. Pre-dilatation was performed in 62 patients (65.3%), and post-dilatation in 32 patients (33.7%). Most procedures were performed percutaneously (63.1%) through the right femoral route (88.4%). The mean depth of implantation was 3.59 ± 1.38 mm. The operative characteristics have been listed in Table 4.

**Table 4:** Operative Data of the Studied Patients (N = 95)

Variables	Results
DI (mm)	3.59 ± 1.38
Preimplantation dilatation	62 (65.2%)
Postimplantation dilatation	32 (33.6%)
Used approach	
Percutaneous	60 (63.1%)
Surgical	35 (36.9%)
Access site	
Right femoral	84 (88.4%)
Left femoral	10 (11.6%)
Subclavian	1 (1%)

Data are expressed as mean (SD) and frequency (percentage). DI: depth of implant.

### Procedural and early post-procedural outcomes

Early post-procedural complications are listed in Table 5. Complications occurred in 42.1% of patients, with the vast majority being transient or minor occurrences. Five patients (5.2%) had high-grade AV block requiring PPM. Also, 62 patients (65.2%) exhibited new or worsening conduction abnormalities in the form of new LBBB, prolongation of PR, or widening of QRS. PVL (> grade 2) occurred in 14 patients (14.7%) after valves deployment but was addressed successfully in all cases with postimplantation balloon dilatation. No patient had residual moderate or severe PVL at discharge. Postprocedural aortic regurgitation was low on follow-up echocardiography. Cerebrovascular accident occurred in three patients (3.1%), and clinically relevant hemorrhage in seven patients (7.4%). Acute kidney injury (contrast-induced nephropathy (CIN) stage ≥ 1) occurred in five patients (5.2%), and valve embolization in one patient (1%).

**Table 5:** Complications Among the Studied Patients

Complications	Number of patients
High-grade AV block requiring PPM	5 (5.2%)
CVS	3 (3.1%)
CIN	3 (3.1%)
Stage 1	2
Stage 2, 3	0
Stage 4	1
Hemorrhage	7 (7.36%)
Type 1	6
Type 2	1
Type 3, 4	0
Valve embolization	1 (1%)
Initial paravalvular leak (> 2)	14 (14.7%)
Final paravalvular leak after post dilatation (> 2)	0
Residual immediate MPG (mm Hg, > 10)	7
Residual MPG after dilatation (mm Hg, > 10)	0
Overall complications	40 (42.1%)

CIN: contrast-induced nephropathy; CVS: cerebrovascular stroke; MPG: mean pressure gradient; PPM: permanent pacemaker implantation.

### Association between calcification and clinical outcomes

**Figure 1:**
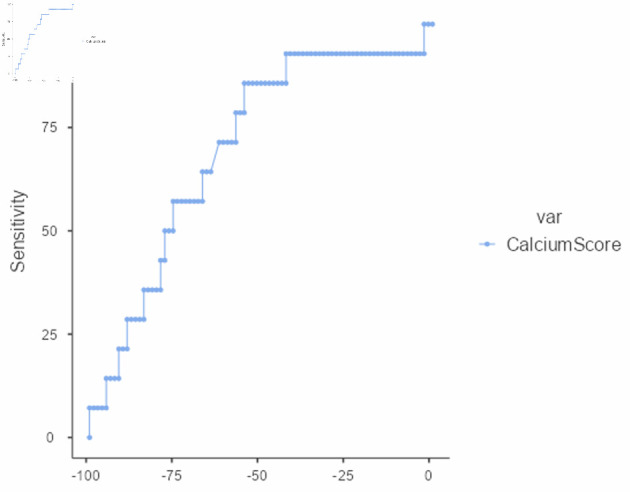
ROC curve for prediction of post-TAVI paravalvular leak using aortic calcium score. ROC: receiver operating characteristic; TAVI: transcatheter aortic valve implantation.

**Figure 2:**
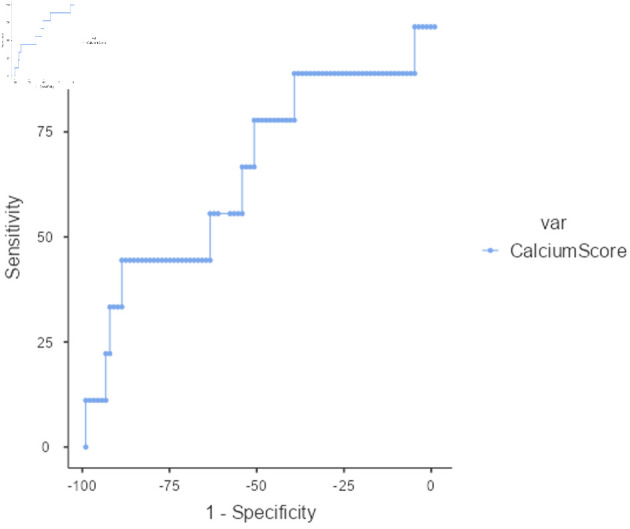
ROC curve for prediction of post-TAVI high-grade AV block using aortic valve calcium score. ROC: receiver operating characteristic; TAVI: transcatheter aortic valve implantation; AV: atrioventricular.

**Figure 3:**
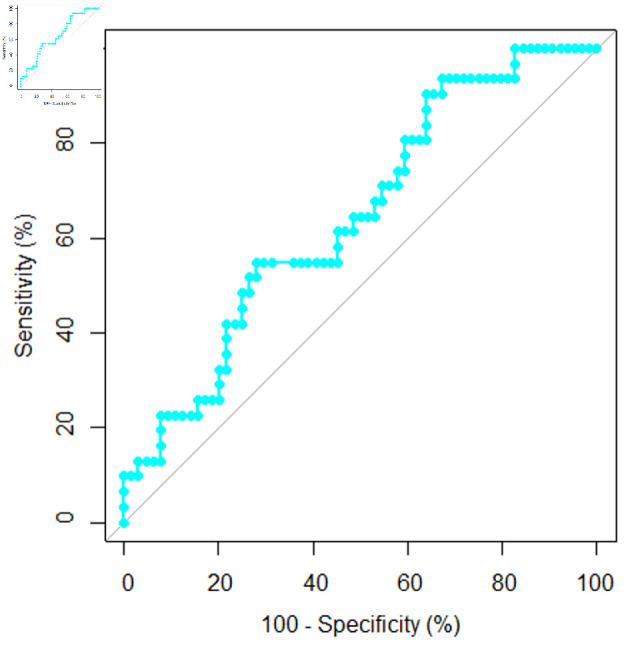
ROC curve for prediction of postimplantation dilatation using the aortic valve calcium score. ROC: receiver operating characteristic.

ROC curve analysis demonstrated that aortic valve calcium score was associated with the occurrence of PVL, high-grade AV block, and the need for postimplantation dilatation. ROC analysis showed an AUC of 0.70 for predicting PVL (cut-off calcium score = 2,206; sensitivity = 85.7%, specificity = 50%), an AUC of 0.66 for predicting high-grade AV block (cut-off = 1,972; sensitivity = 88.9%, specificity = 40.2%), and an AUC of 0.64 for dilatation after implantation (cut-off = 2,900; sensitivity = 54%, specificity = 71%). LVOT calcification was also a highly significant predictor of severe AV block (P = 0.027) in univariate analysis, but not with any other complication such as PVL or post-dilatation. Table 6 and Figures 1–3 summarize these findings.

**Table 6:** Cut-Off Points for Aortic Valve Calcification Measurements as Predictors for Different Study Variables

Variables	Cut-off point	Sensitivity	Specificity	PPV	NPV	AUC
Post-TAVI paravalvular leak						
Calcium score	2,206	85.71%	50%	22.64%	95.35%	0.700
Post-TAVI high grade AV block						
Calcium score	1,972	88.89%	40.23%	97.22%	13.33%	0.660
Postimplantation dilatation						
Calcium score	2,900	54%	71%	79.07%	43.20%	0.64

AUC: area under the curve; AV: atrioventricular; NPV: negative predictive value; PPV: positive predictive value; TAVI: transcatheter aortic valve implantation.

### Mortality and follow-up

Two in-hospital deaths (2.1%) were noted but neither of them was valve-related (one due to cerebrovascular stroke (CVS) and one due to acute renal failure). No further deaths or re-interventions were noted at 1-month follow-up. There were no survivors with moderate and severe regurgitation on echocardiography, and all 30-day survivors were clinically stable with intact valve function.

## Discussion

TAVI is a novel, safer, less invasive procedure that is expanding every day to reach more patients categories due to the huge success in the past two decades in improving the quality of life for hundreds of thousands of patients [9]. In our study, we aimed to assess the role of landing zone calcification (aortic valve leaflets and LVOT calcification) assessed by non-contrast and contrast-enhanced CT scans and its role in predicting TAVI outcomes.

Calcium assessment included quantitative assessment of the calcium score, visual assessment of calcium extension from grade 1 to grade 4, and assessment of the LVOT calcification.

Our study showed that aortic valve calcium score was associated with PVL, high-grade AV block and the need for postimplantation dilatation. Also, our study showed that LVOT calcification was associated with high-grade AV block.

Our study showed that only five (5.2%) patients developed high-grade AV block requiring permanent pacemaker (four patients with complete heart block (CHB), one patient had Mobitz II). It is of note that two of our patients developed intermittent high-grade AV block, which required Holter monitoring to detect.

On the contrary, Fadahunsi et al reported in 2016 that 25.4% to 28.0% of patients who had TAVI using self-expanding Medtronic CoreValve System developed conduction abnormalities requiring permanent pacemaker. The lower rate of patients of PPM implantation observed in our study may be attributed to differences in valve generation, as the older-generation Corevalve needed more predilatation, causing more AV and His bundle compression and edema [10]. Another explanation could be the difference in mean age, as the mean age in the previous study was 84 years, while the mean age in our study was 73 years; this could have affected their conductive system and predisposed patients to high-grade AV block. On the other hand, Abdelshafy et al stated in 2023 that only 15 patients (11.6%) required PPM at 30 days after TAVI procedure using the Evolute R/pro/Pro plus, which is consistent with our results [11].

One of our patients developed paroxysmal atrial fibrillation (AF), which was successfully converted to sinus rhythm after a 300 mg intravenous (IV) amiodarone infusion in the cardiac care unit (CCU), while two other patients developed permanent AF. On the contrary, Ryan et al reported in 2022 that the incidence of new-onset AF after TAVI was 9.9%. The higher incidence could be attributed to the differences in THV generations and implantation techniques, as their study pooled data from 2016 to 2020. In addition, patients in earlier TAVI trials were generally older, and comorbidities such as pulmonary hypertension, severe mitral regurgitation, and the use of transapical approach could have affected their results [12].

We found that aortic valve calcium score had a statistical significance as it had an impact on the risk of having significant PVL after TAVI, which needed post-dilatation to obtain satisfying outcomes. However, after post-dilatation, none of our patients had more than mild PVL. Therefore, in our study, there were no significant PVL. This is consistent with what Uebelacker et al reported in 2023: aortic valve calcification correlated with the incidence of more than mild PVL requiring corrective measures after TAVI, which was observed in 25.7% of patients and decreased to 7% after corrective measures were applied [13].

Also, we found that aortic valve calcium score had a statistical significance as it had an impact on the risk of high-grade AV block post-TAVI. This is consistent with the study of Sharma et al in 2020, who found that leaflet calcification quantified by pre-TAVR CT could be a novel and powerful predictor of pacemaker dependence after TAVR [14].

Moreover, our study showed that LVOT calcium had a significant positive correlation only with CHB (P value = 0.027). This is in contrast with the results reported by Waldschmidt et al in 2022, who found that the rate of PPM post-TAVI was similar in both groups (LVOT calcium group and non LVOT calcium group). However, this could be due to the use of variable platforms for THV (Lotus, Sapien 3, Acurate Neo 2 in this study) [15].

Our study did not show any significance for depth of implantation on the risk of having post-TAVI CHB. On the contrary, Baraka et al reported in 2024 significant results in their study, which was designed to assess the ratio of depth of implantation to the length of the membranous septum. On the contrary, in our study, we only assessed the depth of implantation as predictor for CHB [16]. Although membranous septum length has been consistently identified as a predictor of conduction disturbances after TAVI, no significant association was observed in our cohort. This finding should be interpreted cautiously given the limited number of conduction events and the resulting restricted statistical power.

We also found that aortic valve calcification measurements had no statistical significance on the risk of having post-TAVI CVS events. This goes with most studies, which have stated that cerebrovascular (CV) events occurred in about 2.5% of TAVI procedures. With wide spread of TAVI to more patient categories and the growing number of TAVI, operators kept the CVS incidence near its incidence since 2009. Our study found that only aortic valve calcium score had statistical significance as regard postimplantation balloon dilatation, which was more common in patients with heavy calcification assessed with calcium score. This is consistent with the study of Clerfond et al [17], who found in 2022 that high aortic valvular calcium score, volume and mass assessed by CT scan, were associated with suboptimal prosthesis implantation outcomes in direct TAVI. The difference here could be due to limited number of patients that underwent calcium volume and mass assessment [17].

### Limitations

This study has several limitations that should be considered when interpreting the findings. First, this was a single-center observational study with a relatively small sample size, which may limit statistical power and the generalizability of the results. Second, the number of clinical events was low, particularly high-grade AV block requiring PPM (n = 5). Consequently, the predictive performance of the reported ROC-derived cut-off values should be interpreted with caution, and the possibility of statistical overfitting cannot be excluded.

Third, follow-up was limited to 30 days after TAVI; therefore, the prognostic significance of aortic valve and LVOT calcification for longer-term clinical outcomes could not be evaluated. Fourth, the study population was predominantly treated with the Evolut R valve platform, with only two patients receiving a balloon-expandable valve. Accordingly, the findings may not be directly applicable to other THV systems.

Fifth, LVOT calcification was assessed using a qualitative visual classification rather than quantitative calcium volume or Agatston-based measurements, which may have limited the precision of risk stratification. In addition, detailed regional analysis of calcification distribution, including leaflet-specific (right coronary cusp (RCC), left coronary cusp (LCC), and non-coronary cusp (NCC)) and NCC-LVOT calcification patterns, was not systematically performed, despite evidence suggesting that calcification location may influence post-TAVI outcomes.

Sixth, although membranous septum length and implantation depth were recorded, the limited number of conduction events restricted the ability to comprehensively evaluate their interaction with calcification burden and their relative contribution to post-procedural conduction disturbances.

Finally, the study population was relatively younger and had a lower operative risk profile compared with many contemporary TAVI registries. Moreover, patients who died during hospitalization or were lost to follow-up were not included in the final analysis, introducing the possibility of selection bias. Therefore, the findings should be considered hypothesis-generating and require validation in larger multicenter studies with longer follow-up and higher event rates.

### Conclusions

Calcification at the aortic valve and LVOT calcifications showed some correlation with the development of certain early complications following TAVI, namely conduction disturbances and PVL. This forms a basis for the relevance of a comprehensive analysis through the use of a CT scan before carrying out the procedure. However, since the sample size was small and the events were rare, the findings should be treated as hypothesis generating.

## Data Availability

The data supporting the findings of this study are available from the corresponding author upon reasonable request.

## Abbreviations

AF: atrial fibrillation
AR: aortic regurgitation
AS: aortic stenosis
AUC: area under the curve
AV: atrioventricular
AVA: aortic valve area
BAV: bicuspid aortic valve
BMI: body mass index
BSA: body surface area
CABG: coronary artery bypass grafting
CHB: complete heart block
CIN: contrast-induced nephropathy
CrCl: creatinine clearance
CT: computed tomography
CVS: cerebrovascular stroke
DI: depth of implantation
ECG: electrocardiogram
EF: ejection fraction
EuroSCORE II: European System for Cardiac Operative Risk Evaluation II
LMCA: left main coronary artery
LVEDD: left ventricular end-diastolic diameter
LVESD: left ventricular end-systolic diameter
LVOT: left ventricular outflow tract
LBBB: left bundle branch block
MDCT: multidetector computed tomography
MPG: mean pressure gradient
NPV: negative predictive value
PPG: peak pressure gradient
PPM: permanent pacemaker implantation
PPV: positive predictive value
PVL: paravalvular leak
RBBB: right bundle branch block
RCA: right coronary artery
ROC: receiver operating characteristic
RVSP: right ventricular systolic pressure
SAVR: surgical aortic valve replacement
STS: Society of Thoracic Surgeons
THV: transcatheter heart valve
TTE: transthoracic echocardiography
TAVI: transcatheter aortic valve implantation
VARC-3: Valve Academic Research Consortium-3
